# Silencing of specificity protein 1 protects H9c2 cells against lipopolysaccharide-induced injury via binding to the promoter of chemokine CXC receptor 4 and suppressing NF-κB signaling

**DOI:** 10.1080/21655979.2022.2026548

**Published:** 2022-01-20

**Authors:** Zhao Zhu, Guoxiu Zhang, Dahuan Li, Xiaojun Yin, Tianzhong Wang

**Affiliations:** Department of Emergency, The First Affiliated Hospital, and College of Clinical Medicine of Henan University of Science and Technology, Luoyang, 471003 China

**Keywords:** Septic myocardial injury, G protein-coupled protein receptor cxc chemokine receptor 4, specificity protein 1, nuclear factor kappa B signaling

## Abstract

G protein-coupled protein receptor CXC chemokine receptor 4 (CXCR4) has been shown to be involved in the development of sepsis; however, it remains unclear whether CXCR4 participates in the septic myocardial injury. In our study, treatment with lipopolysaccharide (LPS) increased the expression of specificity protein 1 (SP1) and CXCR4 in H9c2 cells. Notably, a positive association between SP1 and CXCR4 expression was observed in LPS-treated H9c2 cells, and SP1 positively regulated CXCR4 expression in H9c2 cells. Moreover, silencing of SP1 or CXCR4 suppressed LPS-induced inflammation and cell apoptosis in H9c2 cells, as evidenced by the increase in cell viability and decrease in lactate dehydrogenase release, interleukin (IL)-6, IL-8, and tumor necrosis factor (TNF)-α levels, and caspase-3 activity. Additionally, overexpression of CXCR4 abolished the protective effects of SP1 silencing on LPS-induced injury in H9c2 cells. SP1 was also shown to enhance the promoter activity of CXCR4 by directly binding with the binding motif site – 109/–100 in CXCR4 promoter. Besides, downregulation of SP1 or CXCR4 blocked LPS-induced activation of the NF-кB signaling in H9c2 cells. Furthermore, inhibition of NF-кB signaling by DHMEQ abolished LPS-induced myocardial inflammation and apoptosis. In conclusion, silencing of SP1 protected H9c2 cells against LPS-induced injury by binding to the promoter of CXCR4 and suppressing the NF-κB signaling pathway. Hence, our findings provide evidence that manipulation of SP1 or CXCR4 may be an effective approach to promote prevention or recovery of septic myocardial injury, and thereby, may serve as a potential therapeutic strategy for sepsis.

## Introduction

Sepsis is characterized by the systemic inflammatory response syndrome initiated by bacterial infection, with high mortality [1]. Clinically, myocardial dysfunction due to sepsis is regarded as one of the most severe complications in sepsis and septic shock, which is manifested as decreased systolic contractility and impaired diastolic function [2]. Septic myocardial dysfunction is a pivotal factor in the poor clinical outcome of patients with sepsis [3]. Although increasing studies have been paid attention to septic myocardial dysfunction [4–6], the mechanism of septic myocardial injury remains largely unclear, and an effective treatment for septic myocardial injury has not yet been established. Lipopolysaccharide (LPS), also known as endotoxins, is an essential component of the outer membrane of Gram-negative bacteria, which can cause septic myocardial dysfunction by stimulating inflammation and leading to myocardial death [7]. Thus, inhibiting LPS-induced myocardial damage may be a promising therapeutic strategy for septic myocardial injury.

The G protein-coupled protein receptor CXC chemokine receptor 4 (CXCR4), located on chromosome 4, has been reported to participate in multiple biological processes, such as cell proliferation, survival, and angiogenesis, by initiating the activation of its downstream signaling pathways [8,9]. Notably, CXCR4, as an alpha chemokine receptor, can affect the directed migration of leukocytes to sites of inflammation during the development of an inflammatory response [10]. Increasing evidence suggests that CXCR4 acts as a crucial modulator in the development of inflammation-related diseases. Previous studies have reported that the serum level of CXCR4 was much higher in neonates with sepsis than that in neonates without sepsis, indicating the diagnostic value of CXCR4 in sepsis [11,12]. Another study showed that pharmacologic antagonism of CXCR4 with plerixafor improved the survival of mouse with polymicrobial sepsis and reduced sepsis-induced peripheral T cell loss, highlighting the crucial role of CXCR4 in controlling sepsis-induced immune dysfunction and mortality [13]. Additionally, the participation of CXCR4 in the development of myocardial diseases has been previously documented. CXCR4 blockade by POL5551 promoted the mobilization of regulatory T cells, reduced the expression of inflammatory genes, enhanced angiogenesis in the infarcted region, and improved contractile dysfunction after myocardial infarction [14]. Moreover, CXCR4 was considered as a therapeutic target for acute myocardial infarction due to its role in inflammation and progenitor cell recruitment [15]. However, whether CXCR4 plays a role in the development of septic myocardial injury and its underlying mechanisms have not yet been explored.

In this study, we aimed to determine the role of CXCR4 in the development of septic myocardial injury and its up- and down-stream mechanisms. It was found that silencing of CXCR4 ameliorates LPS-induced inflammatory response and apoptosis in H9c2 cells. Regarding the molecular mechanisms, we hypothesized that transcription factor specificity protein 1 (SP1) promotes CXCR4 expression by binding to the promoter of the CXCR4 gene and further activates the NF-кB signaling and thereby functions in the development of septic myocardial injury. Our findings might provide a novel and potential therapeutic strategy for septic myocardial injury.

## Materials and methods

### Cell culture and treatment

Rat cardiac cells (H9c2), obtained from American Type Culture Collection (Manassas, VA, USA), were grown in Dulbecco’s modified Eagle’s medium (DMEM) supplemented with 10% fetal bovine serum (FBS; Solarbio, Beijing, China) and 1% penicillin–streptomycin (Solarbio) in a 5% CO_2_ incubator at 37°C. The medium was refreshed every 2 days. After reaching 80% confluence, H9c2 cells were treated with increasing doses (0, 0.1, 1, 2, 5, and 10 μg/mL) of LPS for 24 h.

### Cell transfection

The full sequence of SP1 or CXCR4 was amplified and then subcloned into pcDNA-3.0 vector to generate pcDNA-SP1 or pcDNA-CXCR4 constructs, respectively. si-SP1, si-CXCR4, and their negative control (si-NC) were obtained from RiboBio (Guangzhou, China). Cell transfection was carried out using Lipofectamine 3000 (Invitrogen, Waltham, MA, USA) following the product manual.

### Quantitative reverse transcription polymerase chain reaction (qRT-PCR)

Total RNA isolation was performed using TRIzol reagent (Qiagen, Duesseldorf, Germany). cDNA synthesis was carried out with the Omniscript RT kit (Qiagen). Next, the expression of CXCR4, SP1, tumor necrosis factor (TNF)-α, interleukin (IL)-8, and IL-6 mRNA was determined with the SYBR® Premix Ex Taq™ Perfect Real-Time kit (Takara, Dalian, China) as per the manufacturer’s instructions. The primer sequences used in this study are summarized in Table 1. The relative expression of genes was calculated according to the 2^–ΔΔCt^ method using GAPDH as a housekeeping gene.

**Table 1.** Sequences of primers used in this study

Gene	Forward primer	Reverse primer	Product length	Accession
CXCR4	5’-GTGGCTGACCTCCTCTTTGT-3’	5’-TGTTGGTGGCGTGGACAAT-3’	180bp	NC_051348.1
SP1	5’-GGCTACCCCTACCTCAAAGG-3’	5’-CACAACATACTGCCCACCAG-3’	103bp	NC_051342.1
TNF-α	5’-ACAAGGCTGCCCCGACTAC-3’	5’-CTCCTGGTATGAAATGGCAAATC-3’	67bp	NC_051355.1
IL-8	5’-CGGAAGGAACCATCTCACTGTG-3’	5’-AGAAATCAGGAAGGCTGCCAAG-3’	77bp	NC_000004.12
IL-6	5’-GTCAACTCCATCTGCCCTTCAG-3’	5’-GGCAGTGGCTGTCAACAACAT-3’	264bp	NC_051339.1
GADPH	5’-CCGTGTTCCTACCCCCAATG-3’	5’-GTCCACCACCCTGTTGCTGTA-3’	480bp	NC_051339.1

### Western blotting

Western blot assay was performed as previously described [9]. Total protein was extracted from H9c2 cells using lysis buffer and then loaded onto 12% sodium dodecyl sulfate-polyacrylamide gels in running buffer before being subjected to electrophoresis. Following this, the samples were transferred to polyvinylidene difluoride membranes. The membranes were blocked and probed with primary antibodies (Novus, Shanghai, China) against CXCR4, SP1, p65, and p-p65 overnight at 4°C. After washing, the membranes were immunoblotted for 1 h at room temperature with secondary antibodies (Novus), and the signal was detected using an ECL kit (Beyotime, Shanghai, China).

### Cell counting kit-8 (CCK-8) assay

H9c2 cells were seeded in a 96-well plate. After treatment, CCK-8 solution (Beyotime) diluted with DMEM was added into each well and incubated in a 5% CO_2_ incubator at 37°C. After 1 h of incubation, H9c2 cells were collected and the optical density at 450 nm was measured.

### Determination of lactate dehydrogenase (LDH) release

LPS-induced myocardial injury was quantitatively measured by the determination of LDH release using the LDH assay kit (Nanjing Jiancheng Bioengineering Institute, Nanjing, China), as per the manufacturer’s recommendations. Briefly, the H9c2 cell supernatant was obtained after centrifugation and incubated with matrix buffer and coenzyme I solution in a water bath at 37°C for 15 min. Next, the samples were incubated with 2,4-dinitrophenylhydrazine solution in a water bath of 37°C for 15 min, followed by treatment with 0.4 mol/L NaOH solution for 3 min at room temperature. Each sample was analyzed at a wavelength of 450 nm with a microplate reader.

### Enzyme-linked immunosorbent assay (ELISA)

The levels of TNF-α, IL-8, and IL-6 were determined with the rat TNF-α ELISA kit (Solarbio), rat IL-8 ELISA kit (Solarbio), and rat IL-6 ELISA kit (Solarbio), respectively, according to the manufacturer’s instructions.

### Detection of apoptosis by terminal-deoxynucleotidyl transferase mediated nick end labeling (TUNEL) staining

Cells were fixed with 4% paraformaldehyde for 15 min and washed 3 times with phosphate buffered saline. Cells were then stained with the TUNEL reaction mixture (Beyotime) for 1 h at 37°C in a moist chamber, according to the manufacturer’s instructions. The nuclei were visualized by incubation with 4’,6-diamidino-2-phenylindole (DAPI; Boster, Wuhan, China). Images were captured with a fluorescence microscope (Nikon Eclipse, Tokyo, Japan).

### Measurement of caspase-3 activity

Cells were fixed with 4% paraformaldehyde for 15 min, and then permeabilized with 0.02% Triton X-100 for 20 min at room temperature. Caspase-3 activity was detected by visualization of caspase-3 via antibodies directly labeled with phycoerythrin (red) (BD Pharmingen, San Diego, CA, USA). Images were acquired with a fluorescence microscope (Nikon Eclipse).

### Chromatin immunoprecipitation (ChIP) assay

The interaction between SP1 and CXCR4 was explored by ChIP assay with the EZ-ChIP kit (Millipore, Billerica, MA, USA) following the product manual. H9c2 cells were crosslinked with 1% formaldehyde at 37°C for 15 min. Following this, H9c2 cells were suspended in lysis buffer and then sonicated on ice to reduce the chromatin length to 200 bp–2 kb. The lysates were diluted using ChIP-dilution buffer. Next, the supernatant was collected and incubated overnight with anti-SP1 antibody (Novus) or anti-IgG antibody (negative control; Novus) at 4°C, followed by precipitation with protein G-agarose. The immunocomplex was then eluted with elution buffer. Thereafter, NaCl solution was added and the precipitated DNA was maintained at 65°C for 4 h to reverse the crosslinking. After digestion with RNase A and proteinase K, the DNA was purified and used as a template for qRT-PCR assay.

### Luciferase reporter assay

Different lengths of the upstream regions of CXCR4 gene were amplified and subcloned into pGL3 luciferase vector to generate CXCR4 promoter constructs. Using a site-direct mutagenesis approach, the SP1-binding motifs (–109 to –100 bp and –89 to –80 bp) were converted to the complementary sequence to eliminate potential SP1 binding, and then, respectively, cloned into pGL3 luciferase vector to generate pGL3-CXCR4(–200/–109/–100)^mut^ and pGL3-CXCR4(–200/–89/–80)^mut^ constructs. All the recombinant constructs were transfected into H9c2 cells with or without pcDNA-NC or pcDNA-SP1 using Lipofectamine 3000, following the manufacturer’s protocol. At 48 h after transfection, H9c2 cells were collected to test the luciferase activity using a dual-luciferase reporter assay system (Promega, Madison, WI, USA), as per the manufacturer’s instructions.

### Statistical analysis

All data were analyzed using the SPSS 20.0 software and are presented as the mean ± standard error. Differences between groups were determined using Student’s *t*-test and one-way analysis of variance. The results were deemed to be statistically significant at *P* < 0.05.

## Results

In this study, we explored the role of CXCR4 and the potential upstream and downstream mechanisms in the development of septic myocardial injury. The data revealed that the expression of CXCR4 was upregulated in H9c2 cells treated with LPS and silencing of CXCR4 ameliorates LPS-induced inflammatory response and apoptosis in H9c2 cells. Mechanistic investigations showed that SP1 enhances CXCR4 expression by binding to its promoter and further activates the NF-кB signaling, thereby contributing to the development of septic myocardial injury.

### SP1 expression is positively correlated with CXCR4 expression in LPS-treated H9c2 cells

**Figure 1.**
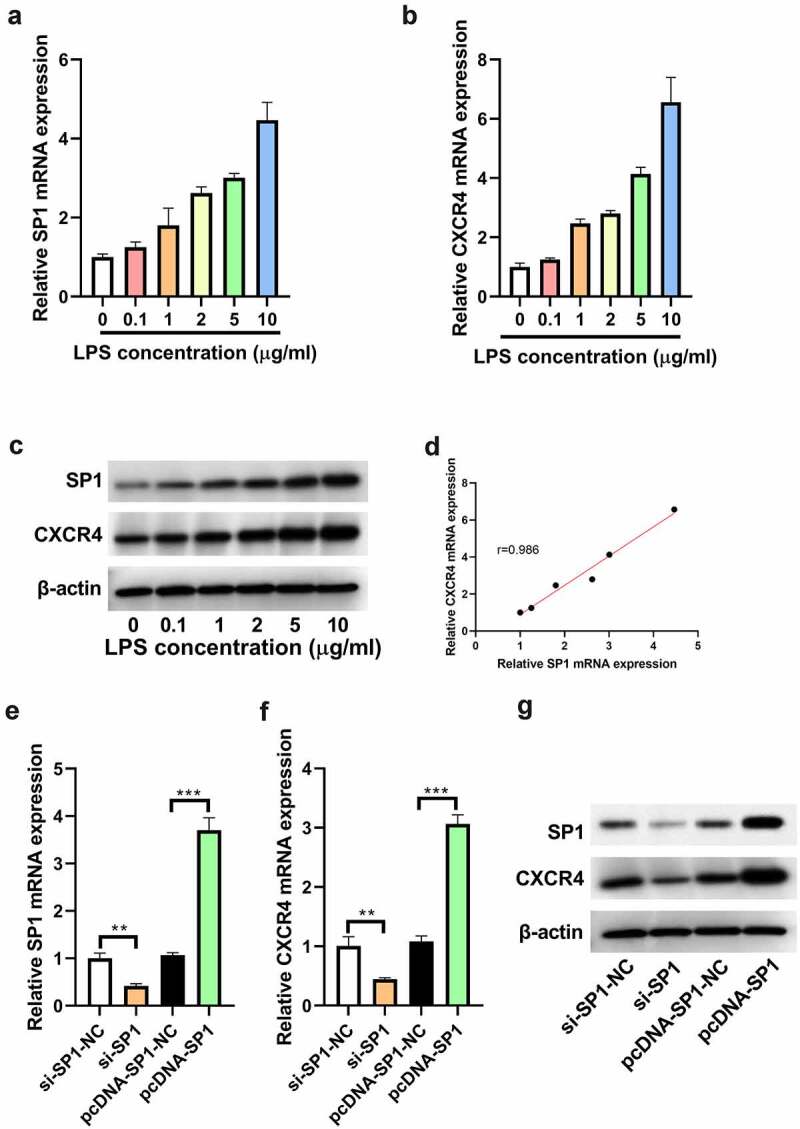
SP1 expression is positively correlated with CXCR4 expression in LPS-treated H9c2 cells. H9c2 cells were exposed to increasing doses (0, 0.1, 1, 2, 5, and 10 μg/mL) of LPS for 24 h. (a and b) qRT-PCR analysis of SP1 and CXCR4 expression in LPS-stimulated H9c2 cells. (c) Western blot analysis of SP1 and CXCR4 expression in LPS-stimulated H9c2 cells. (d) Correlation analysis of SP1 and CXCR4 expression in LPS-treated H9c2 cells. (e and f) qRT-PCR and (g) Western blot analysis of SP1 and CXCR4 expression in H9c2 cells transfected with si-SP1, pcDNA-SP1, or their matched controls. **P < 0.01, ***P < 0.001.

H9c2 cells were exposed to increasing doses (0, 0.1, 1, 2, 5, and 10 μg/mL) of LPS for 24 h and then subjected to qRT-PCR and Western blotting. Results showed that LPS dose-dependently increased the mRNA expression of SP1 and CXCR4 in H9c2 cells (Figure 1a and 1b). Similarly, the protein expression of SP1 and CXCR4 was consistent with their mRNA expression in LPS-treated H9c2 cells (Figure 1c). Notably, SP1 expression was positively correlated with CXCR4 expression in LPS-treated H9c2 cells (Figure 1d). To further investigate the relationship between SP1 and CXCR4, we altered the expression of SP1 in H9c2 cells and then assessed the expression of CXCR4 using qRT-PCR and Western blotting. As expected, the mRNA and protein levels of SP1 were markedly increased in pcDNA-SP1-transfected H9c2 cells, but significantly reduced in si-SP1-transfected H9c2 cells (Figure 1e and 1g). Moreover, knockdown of SP1 reduced while forced expression of SP1 elevated the mRNA and protein levels of CXCR4 in H9c2 cells (Figure 1f and 1g).

### Silencing of SP1 or CXCR4 ameliorates LPS-induced inflammatory response and apoptosis in H9c2 cells

**Figure 2.**
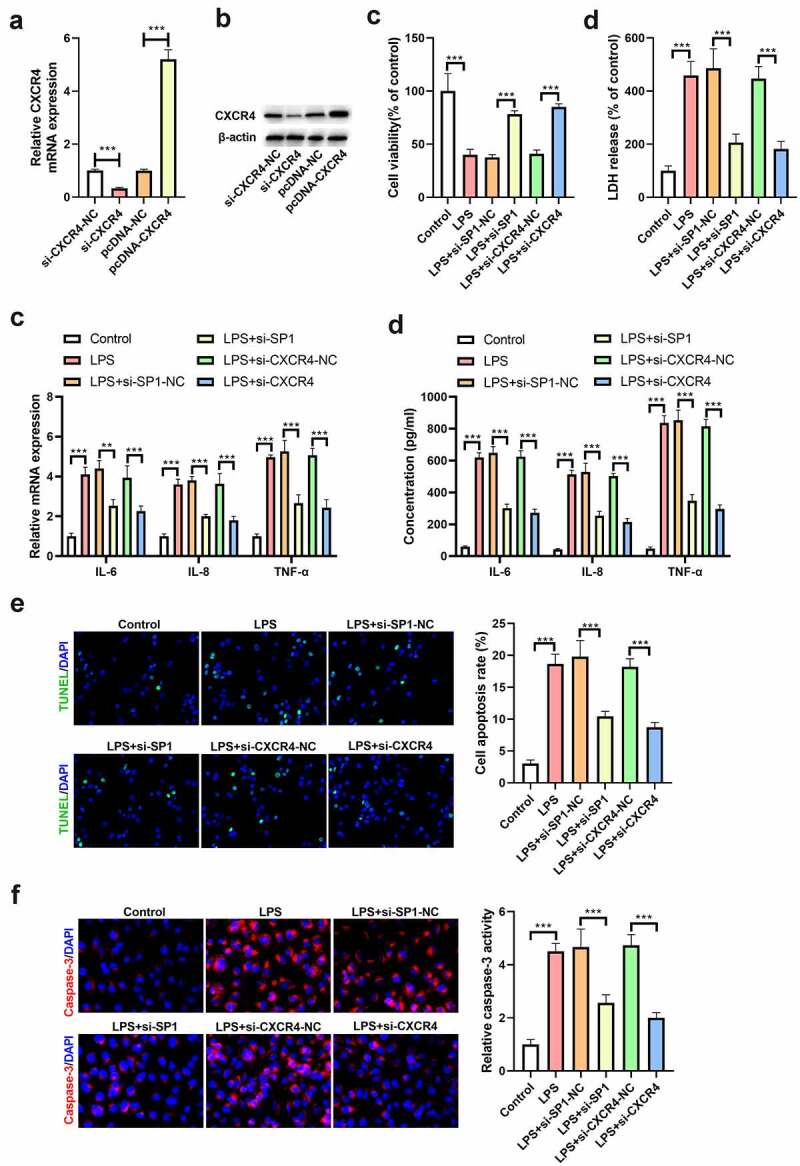
Silencing of SP1 or CXCR4 ameliorates LPS-induced inflammatory response and apoptosis in H9c2 cells. (a) qRT-PCR and (b) Western blot analysis of CXCR4 expression in H9c2 cells transfected with si-CXCR4, pcDNA-CXCR4, or their matched controls. H9c2 cells were transfected with si-SP1, si-CXCR4, or their corresponding controls, and then treated with 10 μg/mL of LPS for 24 h. (c) Cell viability was examined using CCK-8 assay. (d) The release of LDH was examined using a commercial kit. (e) qRT-PCR and (f) ELISA assays were performed to determine the levels of TNF-α, IL-8, and IL-6 in H9c2 cells. (g) TUNEL staining was carried out to evaluate the apoptosis of H9c2 cells. (h) The activity of caspase-3 was measured to assess cell apoptosis. ***P < 0.001.

To study the roles of SP1 and CXCR4 in septic myocardial injury, H9c2 cells were transfected with si-SP1, si-CXCR4 or their matched controls. The efficiency of cell transfection is shown in Figure 2a and 2b. At 48 h post-transfection, the cells were collected for the assessment of SP1 and CXCR4 mRNA and protein expression, and the results confirmed successful establishment of the cells with downregulation and upregulation of SP1 or CXCR4 (Figure 2a and 2b). Then, these transfected cells were treated with 10 μg/mL LPS for 24 h. As determined by CCK-8 assay, LPS treatment markedly reduced the viability of H9c2 cells, which was undermined following si-SP1 or si-CXCR4 transfection (Figure 2c). In line with this, LDH release was increased in LPS-treated H9c2 cells; however, this effect was blocked by silencing of SP1 or CXCR4 (Figure 2d). In parallel, administration of LPS resulted in elevation of TNF-α, IL-8, and IL-6 levels, as shown by qRT-PCR. However, either SP1 or CXCR4 knockdown attenuated LPS-induced increase of TNF-α, IL-8, and IL-6 levels (Figure 2e). Moreover, the results of qRT-PCR were confirmed by ELISA (Figure 2f). Besides, we also found that LPS treatment induced H9c2 cell apoptosis, and this induction was abolished by downregulation of SP1 or CXCR4 (Figure 2g). Similarly, the activity of caspase-3 was increased following LPS treatment, and silencing of SP1 or CXCR4 suppressed this effect (Figure 2h).

### Overexpression of CXCR4 abolishes the protective effects of SP1 silencing LPS-induced injury in H9c2 cells

**Figure 3.**
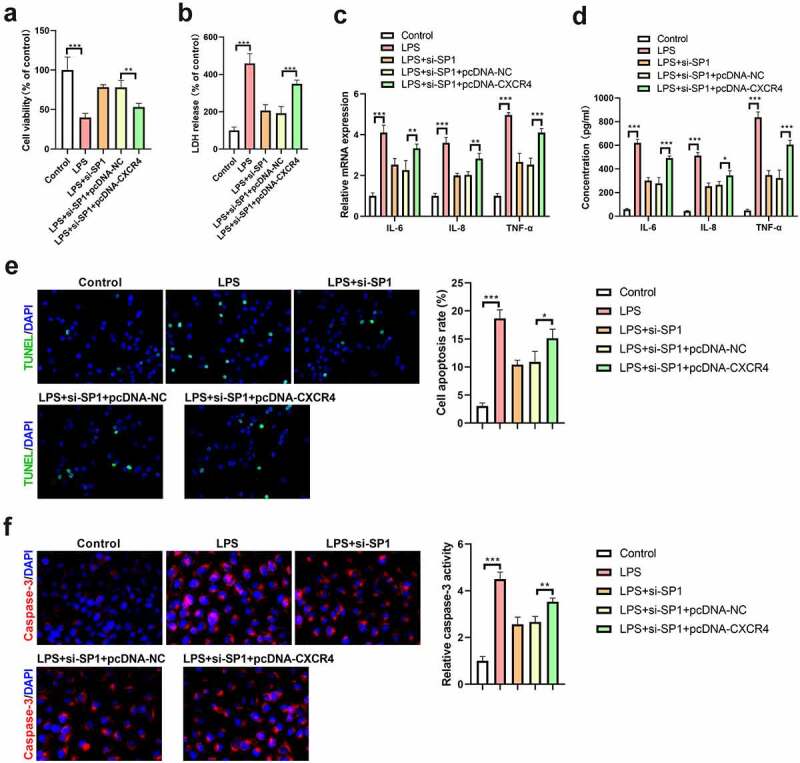
Overexpression of CXCR4 abolishes the protective effects of SP1 silencing on LPS-induced injury in H9c2 cells. H9c2 cells were transfected with si-SP1 alone or together with pcDNA-CXCR4, and then treated with 10 μg/mL of LPS for 24 h. (a) Cell viability was examined using CCK-8 assay. (b) The release of LDH was examined using a commercial kit. (c) qRT-PCR and (d) ELISA assays were performed to determine the levels of TNF-α, IL-8, and IL-6 in H9c2 cells. (e) TUNEL staining was used to evaluate the apoptosis of H9c2 cells. (f) The activity of caspase-3 was measured to assess cell apoptosis. *P < 0.05, **P < 0.01, ***P < 0.001.

To affirm whether the effect of SP1 on LPS-induced injury in H9c2 cells was mediated by CXCR4, H9c2 cells were transfected with si-SP1 alone or together with pcDNA-CXCR4 and then treated with 10 μg/mL of LPS for 24 h. Results of CCK-8 assay showed that silencing of SP1 mitigated LPS-induced inhibition of cell viability, and this effect was abolished by overexpression of CXCR4 (Figure 3a). Moreover, knockdown of SP1 inhibited the release of LDH in LPS-treated H9c2 cells; however, this effect was blocked by pcDNA-CXCR4 transfection (Figure 3b). Meanwhile, downregulation of SP1 restrained LPS-induced increase in TNF-α, IL-8, and IL-6 levels in H9c2 cells, but this effect was abrogated following transfection with pcDNA-CXCR4 (Figure 3c and 3d). Additionally, the inhibitory effect of SP1 silencing on LPS-induced cell apoptosis was blocked by upregulation of CXCR4, as evidenced by the increased rate of cell apoptosis and increased activity of caspase-3 (Figure 3e and 3f).

### SP1 can directly bind to the promoter region of CXCR4 and the key promoter region (–200/–1) of CXCR4 contains the SP1 response element

Using information in the JASPAR database, we found that the promoter region of CXCR4 from different species (human, mouse, and rat) harbors putative SP1 binding sites (Table 2). To investigate whether SP1 activates the transcription of CXCR4 by binding to its putative promoter region, ChIP assay coupled with qRT-PCR was performed. According to the score in the JASPAR database, the following eight top potential binding sites were chosen for ChIP assay: –109 to –100 bp, –318 to –309 bp, –319 to –310 bp, –982 to – 973bp, –1584 to –1575 bp, –1585 to –1576 bp, –89 to –80 bp, and –1089 to –1080 bp.

**Table 2.** The predicted binding sites between SP1 and the CXCR4 promoter region of the corresponding species by the Jaspar database

Species	Matrix ID	Score	Predicted sequence	Start	End
Human	MA0079.4	11.5982	TCGGCCCCGCCCCAC	−1228	−1214
		8.74515	TCCGCCCCGCCCCTG	−135	−121
		7.88218	TCCGCCCCGCCCAGT	−1083	−1069
		6.79744	CAAGTCACTCCCCTT	−388	−374
		2.14913	CCCACCCCGCCTTCT	−90	−76
		1.78601	CCCGCCCCACCCAGT	−1204	−1190
		1.57732	GAATCCCTGCCCATC	−966	−952
		1.22815	CAAGCCGCGCACCTC	−150	−136
		0.923761	TGACCCCCACCCCCA	−1099	−1085
		0.90512	CCCACTCCGCCCCGC	−1088	−1074
Rat	MA0079.2	14.6403	CCCCGCCCCC	−109	−100
		11.8365	CCCCTCCCCA	−318	−309
		11.3147	CCCCCTCCCC	−319	−310
		10.0159	CCACGCCCCC	−982	−973
		9.64527	CCCAGCCCCT	−1584	−1575
		8.82453	CCCCAGCCCC	−1585	−1576
		8.75667	TCCCGCCCCT	−89	−80
		8.57001	TCCCTCCCCG	−1089	−1080
		8.35663	CGCCGCCCCG	−65	−56
		8.32382	CCCCGCCTTA	−1084	−1075
		7.76905	CTCCTCCCGC	−93	−84
		7.50453	CCCCTCCGAG	−84	−75
		7.48874	CCCCTCTACC	−1579	−1570
		7.45529	CACCCCCTCC	−321	−312
		7.24262	CCGCCCCTCC	−87	−78
		7.19922	CTCCCGCCCC	−90	−81
		6.75342	CCCTCCCCGC	−1088	−1079
		6.75342	CCCGCCCCTC	−88	−79
Mice	MA0079.2	14.6403	CCCCGCCCCC	−96	−87
		10.4176	CCCCATCCCC	−313	−304
		10.0159	CCACGCCCCC	−922	−913
		9.4586	CCCATCCCCA	−312	−303
		8.7923	CTCCCTCCCC	−1030	−1021
		8.77275	CCCCGCCTAG	−1024	−1015
		8.75667	TCCCGCCCCG	−76	−67
		8.67041	CCCTTTCTCC	−1914	−1905
		8.57001	TCCCTCCCCG	−1029	−1020
		8.35663	CGCCGCCCCG	−51	−42
		7.9847	CACCACCCCC	−1107	−1098
		7.80555	CCCTGCGCCC	−103	−94
		7.76905	CTCCTCCCGC	−80	−71
		7.50644	CCCCACCCTT	−1042	−1033
		7.264	CCCAGGCTCC	−1132	−1123
		7.19922	CTCCCGCCCC	−77	−68
		7.11887	CGCCCCCGCC	−93	−84
		6.75342	CCCTCCCCGC	−1028	−1019

**Figure 4.**
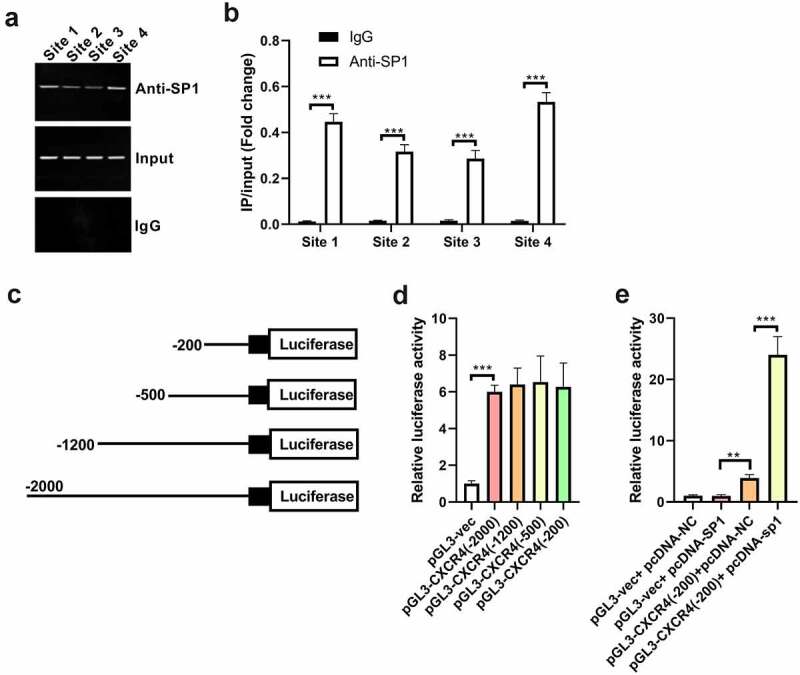
SP1 can directly bind to the promoter region of CXCR4 and the key promoter region (–200/–1) of CXCR4 contains the SP1 response element. (a) ChIP assay was carried out to verify the putative binding sites of SP1 on the promoter region of CXCR4 in H9c2 cells transfected with pcDNA-SP1, using IgG as a negative control. (b) qRT-PCR analysis was performed to quantify the results of ChIP assay. (c) Four pGL3 luciferase vectors containing different sequence deletions of the 5ʹ-flanking region upstream of CXCR4 were generated and named pGL3-CXCR4 (–2000), pGL3-CXCR4 (–1200), pGL3-CXCR4 (–500), and pGL3-CXCR4 (–200). (d) H9c2 cells were transfected with pGL3 vector, pGL3-CXCR4 (–2000), pGL3-CXCR4 (–1200), pGL3-CXCR4 (–500), or pGL3-CXCR4 (–200), and then tested for relative luciferase activity 48 h after transfection. (e) H9c2 cells were co-transfected with pGL3 vector or pGL3-CXCR4 (–200) and pcDNA-SP1 or pcDNA-NC, and then analyzed for relative luciferase activity 48 h after transfection. **P < 0.01, ***P < 0.001.

Since the minimum amplicon size for each transcription factor binding site is ~150 bp for qRT-PCR, it is technically difficult to amplify –109 to –100 bp and –89 to –80 bp separately. Hence, –109 to –100 bp together with –89 to –80 bp regions were amplified as one binding region and termed site 1 in our study. Analogously, –318 to –309 bp together with –319 to –310 bp regions were amplified as site 2, –982 to –973 bp together with – 1089 to –1080 bp regions were amplified as site 3, and –1584 to –1575 bp together with –1585 to –1576 bp regions were amplified as site 4. Results showed that SP1 could bind to site 1, site 2, site 3, and site 4 sequences within the promoter region of CXCR4 in H9c2 cells (Figure 4a). These findings were also verified by quantitative ChIP assay (Figure 4b).

To validate the most potential promoter region of CXCR4, we constructed four pGL3 luciferase vectors containing different sequence deletions of the 5ʹ-flanking region upstream of CXCR4. We fused –2000 bp to –1 bp, –1200 to –1 bp, –500 bp to –1 bp, and –200 to –1 bp of the 5ʹ-flanking region upstream of CXCR4 into pGL3 vector and named them pGL3-CXCR4 (–2000), pGL3-CXCR4 (–1200), pGL3-CXCR4 (–500), and pGL3-CXCR4 (–200), respectively (Figure 4c). As shown in Figure 4d, insertion of the promoter region of CXCR4 markedly increased the relative luciferase activity of pGL3 vectors. Moreover, constructs with –2000 bp, –1200 bp, or –500 bp upstream of the CXCR4 gene presented similar promoter activity as compared to that with –200 bp, revealing the most potential promoter-like sequence in the –200 bp to –1 bp region (Figure 4d).

To further identify the existence of the SP1 response element in the key promoter region of CXCR4, H9c2 cells were co-transfected with pGL3 vector or pGL3-CXCR4 (–200) and pcDNA-SP1 or pcDNA-NC. Results showed that the CXCR4 promoter construct with – 200 bp increased the relative luciferase activity in H9c2 cells, which was further reinforced by upregulation of SP1 (Figure 4e). This finding indicated that the –200 bp to –1 bp region upstream of CXCR4 is a functional promoter region activated by SP1.

### SP1 enhances the promoter activity of CXCR4 by directly binding with the binding motif site –109/–100 in the CXCR4 promoter

The –200 bp to –1 bp region had been verified to be the functional promoter region of CXCR4 and be responsive to SP1. Moreover, the –200 bp to –1 bp region upstream of the CXCR4 gene was found to contain the following two putative SP1-binding motifs based on the JASPAR database: –109 to –100 bp and −89 to −80 bp. These binding motifs were selected to further explore whether the promoter activation of CXCR4 by SP1 is through the direct binding of the SP1-binding motifs in its promoter.

**Figure 5.**
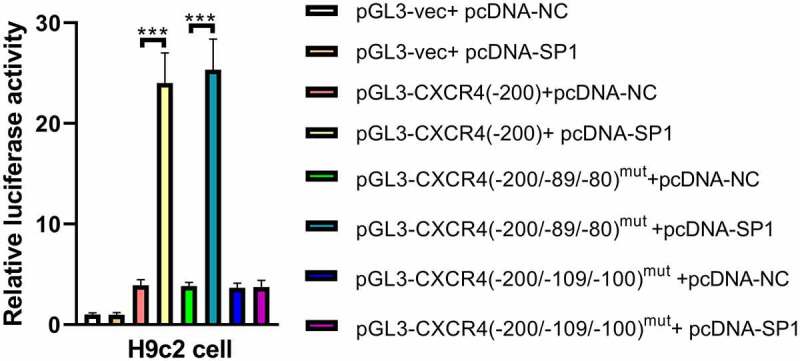
SP1 enhances the promoter activity of CXCR4 by direct binding of SP1 with binding motif site – 109/–100 in CXCR4 promoter. CXCR4 promoter activity was evaluated by performing luciferase reporter assay upon mutation of the SP1-binding motifs (–109 to – 100 bp and – 89 to – 80 bp) in the presence of pcDNA-SP1 or pcDNA-NC in H9c2 cells. ***P < 0.001.

Using a site-directed mutagenesis approach, the identified SP1-binding motifs (–109 to –100 bp and – 89 to – 80 bp) were converted to the complementary sequence to eliminate potential SP1 binding and then, respectively, cloned into pGL3 luciferase vectors to generate pGL3-CXCR4(–200/–109/–100)^mut^ and pGL3-CXCR4(–200/–89/–80)^mut^ constructs. H9c2 cells were co-transfected with pGL3 vector, pGL3-CXCR4 (–200), pGL3-CXCR4 (–200/–109/–100)^mut^, or pGL3-CXCR4 (–200/–89/–80)^mut^, and pcDNA-SP1 or pcDNA-NC. The results showed that upregulation of SP1 strikingly increased the luciferase activity of pGL3-CXCR4 (–200) and pGL3-CXCR4(–200/–89/–80)^mut^ vectors, whereas mutation of the –109/–100 motif completely abolished the reinforcing effect of SP1 on the promoter activity of CXCR4 in H9c2 cells. These data indicate that SP1 enhanced the promoter activity of CXCR4 by directly binding with the binding motif site – 109/–100 in the CXCR4 promoter (Figure 5).

### The NF-κB signaling pathway is involved in mediating the effects of SP1/CXCR4 axis on LPS-induced H9c2 cell injury

**Figure 6.**
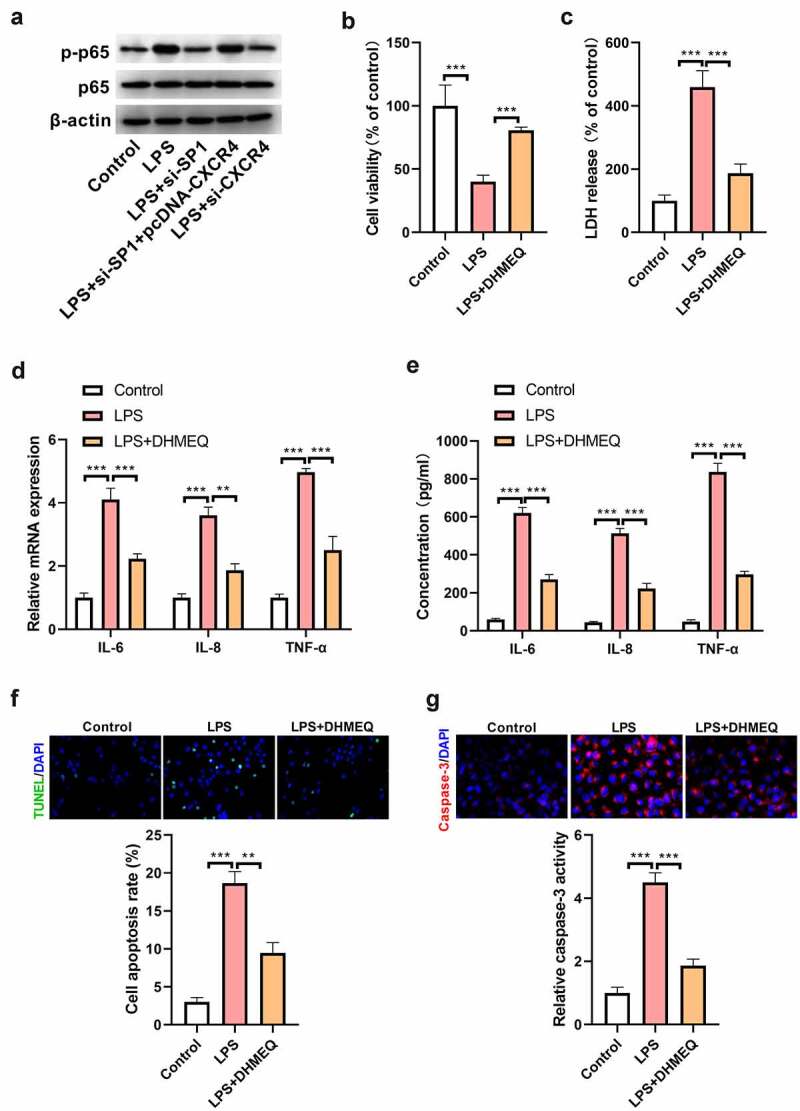
The NF-κB signaling pathway is involved in mediating the effects of SP1/CXCR4 axis on LPS-induced H9c2 cell injury. (a) H9c2 cells were transfected with si-SP1, pcDNA-CXCR4, or si-CXCR4 alone or in combination, and then treated with (10 μg/mL) LPS for 24 h. Western blotting was carried out to evaluate the expression of p-p65 and p65 in H9c2 cells. (b) H9c2 cells were treated with (10 μg/mL) LPS alone or together with (10 μg/mL) DHMEQ, and then subjected to CCK-8 assay. (c) The release of LDH was examined in H9c2 cells stimulated with LPS alone or together with DHMEQ. (d) qRT-PCR and (e) ELISA assays were performed to determine the levels of TNF-α, IL-8, and IL-6 in H9c2 cells stimulated with LPS alone or together with DHMEQ. (f) TUNEL staining was carried out to evaluate the apoptosis of H9c2 cells stimulated with LPS alone or together with DHMEQ. (g) The activity of caspase-3 was measured to assess the apoptosis of H9c2 cells stimulated with LPS alone or together with DHMEQ. ***P < 0.001.

To investigate whether the NF-κB signaling pathway is involved in mediating the effects of the SP1/CXCR4 axis on LPS-induced cell injury, H9c2 cells transfected with si-SP1, pcDNA-CXCR4, or si-CXCR4 alone or in combination were treated with LPS for 24 h and then assayed for p-p65 and p65 expression using Western blot analysis. Results showed that the protein level of p-p65 was increased in LPS-treated H9c2 cells; however, this effect was markedly suppressed by knockdown of SP1 or CXCR4. Furthermore, the inhibitory effect of SP1 silencing on LPS-induced increase of p-p65 levels was blocked by upregulation of CXCR4. However, the protein level of p65 remained unaltered in H9c2 cells treated with LPS, si-SP1, pcDNA-CXCR4, or si-CXCR4 (Figure 6a). Subsequently, we found that DHMEQ (NF-κB inhibitor; 10 μg/mL; MedChem Express, New Jersey, NJ, USA) administration abrogated LPS-induced inhibition of H9c2 cell viability (Figure 6b). Similarly, LPS-induced LDH release was inhibited by DHMEQ treatment (Figure 6c). Administration of DHMEQ also counteracted the increase of TNF-α, IL-1β, and IL-6 levels in LPS-treated H9c2 cells, as demonstrated by qRT-PCR and ELISA (Figure 6d and 6e). Besides, inhibition of NF-κB signaling by DHMEQ treatment resulted in decreased cell apoptosis and caspase-3 activity in LPS-treated H9c2 cells (Figure 6f and 6g).

## Discussion

Myocardial inflammation, characterized by an imbalance of pro- and anti-inflammatory factors, has been implicated to play a key role in the pathophysiology of septic myocardial dysfunction [16]. The imbalance between pro- and anti-inflammatory factors leads to cardiomyocyte death and myocardial microlesions, eventually resulting in myocardial dysfunction [17]. Studies have reported that reducing myocardial inflammation could mitigate septic myocardial dysfunction, which emphasizes the vital role of inflammation in the treatment of septic myocardial dysfunction [18]. As a key modulator of inflammatory response, CXCR4 has been shown to play a critical role in the development of cardiac diseases [19,20]. In mice with dilated cardiomyopathy, pharmacologic antagonism of stromal cell-derived factor-1/CXCR4 signaling with AMD3100 was shown to decrease splenic CD4 + T cell abundance and cardiac fibrosis and improve diastolic and systolic myocardial performance [21]. Moreover, pharmacologic antagonism of CXCR4 with candesartan was shown to reduce diabetes-induced cardiac fibrosis [22]. However, little is known about the functional role of CXCR4 in septic myocardial injury. In this study, we found that LPS treatment increased the expression of CXCR4 in H9c2 cells in a dose-dependent manner. Moreover, silencing of CXCR4 suppressed LPS-induced inflammation and apoptosis in H9c2 cells, indicating the significance of CXCR4 in septic myocardial injury.

The significance of CXCR4 in septic myocardial dysfunction is well documented; however, the mechanism responsible for CXCR4 upregulation in septic myocardial dysfunction is largely unknown. Studies have shown that dysregulation of genes is triggered by multiple mechanisms, in which altered transcription factor activity plays a major role [23]. Here, we identified nine top potential transcription factors that bind to the promoter region of CXCR4 based on the UCSC database. Among them, SP1, a ubiquitously expressed transcription factor, was the only transcription factor that was dose-dependently upregulated by LPS. SP1 has been reported to be involved in various cellular processes by activating the transcription of multiple genes that harbor putative CG-rich Sp-binding sites in their promoter regions [24–26]. For example, SP1 facilitated STK39 expression by binding to its promoter and promoted the proliferation, migration, invasion and epithelial-mesenchymal transition of hepatocellular carcinoma cells [25]; SP1 interacted with ZFPM2-AS1 promoter to transcriptionally activate ZFPM2-AS1 expression and aggravated glioma progression [26]. In addition, our results showed that SP1 expression was positively correlated with CXCR4 expression in LPS-treated H9c2 cells, indicating that SP1 may participate in CXCR4-mediated septic myocardial injury. Recently, SP1 has been documented to serve as a key player in the development of various human diseases, including inflammation-related diseases [27]. For example, silibinin was shown to restrain cigarette smoke condensate-induced airway inflammation in H9c2 cells by inhibiting the phosphorylation of extracellular signal-regulated kinase and the expression of SP1 [28]. A previous study by Lv *et al*. demonstrated that silencing of SP1 attenuated sevoflurane-induced neuroinflammation and apoptosis by activating the cholinergic anti-inflammatory pathway *in vivo* [29]. Notably, SP1 was reportedly implicated in the development of cardiac diseases [30]. For instance, the extract of Dendropanax morbifera was shown to markedly reduce the enlargement of H9c2 cells treated with isoproterenol via suppressing SP1/GATA4-induced natriuretic peptide upregulation [31]. Furthermore, knockdown of SP1 was reported to restrain Ang-II-induced cardiac hypertrophy by regulating the ceRNA network of SNHG14/miR-322-5p/miR-384-5p/PCDH17 [32]. However, the potential role of SP1 in septic myocardial injury remains unclear. In our study, silencing of SP1 ameliorated LPS-induced inflammatory response and apoptosis in H9c2 cells, revealing the important role of SP1 in septic myocardial injury. Remarkably, SP1 was previously shown to be involved in regulating CXCR4 expression. Peritoneal cavity lavage fluid promoted the formation of spheroid, which, in turn, promoted niche-directed metastasis by increasing the expression of CXCR4 in an SP1-dependent manner [33]. However, mechanisms by which SP1 regulates CXCR4 expression remain poorly understood. Herein, we found that SP1 enhanced the promoter activity of CXCR4 by directly binding with the binding motif site – 109/–100 in CXCR4 promoter. More importantly, overexpression of CXCR4 abolished the protective effects of SP1 silencing on LPS-induced injury in H9c2 cells, indicating that SP1 protected H9c2 cells against LPS-induced injury by binding to the CXCR4 promoter.

Although the significance of the SP1/CXCR4 axis in septic myocardial injury was elucidated in our study, its downstream signaling events remain obscure. NF-кB, an inducible transcription factor, participates in various cellular processes, including cell apoptosis and inflammatory response. Increasing evidences suggest that NF-кB signaling is associated with the development of cardiac diseases [34,35]. Upregulation of NF-κB was reported in H9c2 cells stimulated with LPS, and NF-κB mediated the cardioprotective effect of growth factor independence 1 against LPS-induced myocardial inflammation and apoptosis [36]. In LPS-induced sepsis mice, lncRNA-HOTAIR reduced TNF-α expression by suppressing the activation of NF-кB signaling, indicating the involvement of NF-кB signaling in sepsis [37]. Notably, it was previously suggested that CXCR4 is involved in the regulation of NF-кB signaling. For example, stromal cell-derived factor-1/CXCR4 axis reportedly induced the apoptosis of human degenerative nucleus pulposus cells by promoting the activation of NF-кB signaling [38]. In addition, downregulation of CXCR4 was shown to restrain the expression of IL-6 and TNF-α in macrophages by repressing the activation of MAPK and NF-κB signaling [39]. Interestingly, SP1 has been reported to be involved in some diseases via direct interaction with NF-κB. For example, an interaction between the DNA-binding domains of p65 and SP1 mediated human immunodeficiency virus gene activation [40]. In addition, tolfenamic acid and dietary spice curcumin treatment enhanced the anti-proliferative effects in pancreatic cancer cells via suppressing SP1 expression, disrupting NF-kB translocation to the nucleus and cell cycle phase distribution [41]. Notably, NF-кB is an important signaling pathway by which SP1 or CXCR4 functions. In this study, treatment of H9c2 cells with LPS induced the activation of NF-кB signaling, which was blocked by downregulation of SP1 or CXCR4. Besides, overexpression of CXCR4 mitigated the inhibitory effect of SP1 silencing on LPS-induced NF-кB signaling activation in H9c2 cells. Furthermore, inhibition of NF-кB signaling by DHMEQ abolished LPS-induced myocardial inflammation and apoptosis. These data indicate that the SP1/CXCR4 axis regulated LPS-induced myocardial inflammation and apoptosis by controlling the activation of the NF-κB signaling pathway. Whether SP1 functions via direct or indirect regulation of NF-κB may be dependent on the cell type. However, the underlying mechanism by which CXCR4 regulates the NF-kB pathway needs further exploration.

## Conclusion

In summary, our findings showed that SP1 and CXCR4 levels were dose-dependently upregulated in LPS-treated H9c2 cells. Functionally, silencing of SP1 or CXCR4 attenuated LPS-induced myocardial inflammation and apoptosis. Mechanically, silencing of SP1 protected H9c2 cells against LPS-induced injury by binding to the promoter of CXCR4 and suppressing the activation of NF-κB signaling. Hence, our data suggest that manipulation of SP1 or CXCR4 may be an effective approach to promote the prevention or recovery of septic myocardial injury, and thereby, serve as a potential therapeutic strategy for sepsis.

## Data Availability

The data sets used and/or analyzed during the current study are available from the corresponding author on reasonable request.

## Highlights

1. The level of SP1 and CXCR4 was increased in LPS-treated H9c2 cells.
2. Silencing of SP1 or CXCR4 ameliorated LPS-induced H9c2 cell injury.
3. CXCR4 mediated the effects of SP1 silencing on LPS-induced H9c2 cell injury.
4. SP1 enhanced the promoter activity of CXCR4.
5. DHMEQ attenuated LPS-induced H9c2 cell injury.
